# Yoga as a complementary intervention for polycystic ovary Syndrome management: a systematic review

**DOI:** 10.3389/frph.2026.1753608

**Published:** 2026-06-03

**Authors:** Rohit Gautam, Pratibha Maan, Amit Arora, Abilash Nair, Ashraf Ganie, Jayakumari Chellamma, Jayasree Leelamma, Puthiyaveettil Khadar Jabbar, Taruna Arora

**Affiliations:** 1 Division of Reproductive Child Health and Nutrition, Indian Council of Medical Research, New Delhi, India; 2 Department of Rehabilitation Medicine, Sir Ganga Ram Hospital, New Delhi, India; 3 Department of Endocrinology, Government Medical College, Thiruvananthapuram, Kerala, India; 4 Department of Endocrinology, Sher-i-Kashmir Institute of Medical Sciences, SKIMS, Srinagar, Jammu & Kashmir, India; 5 Department of Emergency Medicine, Government Medical College, Thiruvananthapuram, Kerala, India; 6 Department of Radio Diagnosis, Government Medical College, Thiruvananthapuram, Kerala, India

**Keywords:** complementary therapy, Meditation, Mindfulness-based therapy, non-pharmaceutical, Polycystic Ovary Syndrome, Yoga

## Abstract

**Background:**

Polycystic Ovary Syndrome (PCOS), a prevalent metabolic and reproductive disorder, significantly impacts women of reproductive age. The conventional approach offers various symptomatic pharmacological interventions for PCOS, but some of them have adverse effects too. In this context, yoga has emerged as a promising non-pharmacological complementary approach.

**Aim:**

This systematic review aims to explore the therapeutic potential of yoga for managing PCOS with a focus on anthropometric, metabolic, endocrine, and psychological outcomes.

**Methods:**

A systematic search was conducted across three databases i.e., PubMed, Web of Science and Scopus on 17.1.2025. Screening of articles was performed by two authors in two step process. Risk of Bias (RoB) of each study was assessed using Cochrane Risk of Bias version 2.0 (RoB 2). Due to high heterogeneity among studies in terms of type of intervention, duration, outcomes measured etc., meta-analysis could not be performed.

**Results:**

Of the 303 studies initially identified, 9 randomized controlled trials (RCTs) met the inclusion criteria for the systematic review. However, four of these were conducted by the same research group and were considered as a single study in the analysis. Yoga interventions, such as asanas (physical postures) and pranayama (breathing exercises), demonstrated improvement in PCOS symptoms including anthropometric (weight, BMI, hip circumference), metabolic (insulin resistance, serum insulin, fasting blood glucose and lipid profile), endocrine (hirsutism, free testosterone, Anti-mullerian hormone, Luteinizing hormone, Dehydroepiandrosterone etc.), menstrual and psychological outcomes. Meditation and mindfulness-based interventions may help to improve mainly psychological symptoms such as body image, stress, anxiety, depression and quality of life etc. However, it is important to note that there were very few number of studies, that too with lots of heterogeneity, low sample size, diverse outcomes; therefore generalizability of this evidence are limited.

**Conclusion:**

Yoga is a promising non-pharmacological complementary intervention for PCOS management that may offer diverse benefits for anthropometric, endocrine, metabolic and psychological health. However, further clinical trials with robust protocol, large sample size and standardized yoga protocol are essential to establish its long-term efficacy and integration into routine PCOS care.

**Systematic Review Registration:**

https://www.crd.york.ac.uk/PROSPERO/view/CRD420261286708, identifier CRD420261286708.

## 1 Introduction

Polycystic Ovary Syndrome (PCOS) is one of the most common endocrine and metabolic disorders in women of reproductive age. The prevalence of PCOS ranges from 5% to 20%, depending on the diagnostic criteria employed (Rotterdam/NIH/AES) (1). As per Rotterdam criteria, PCOS is characterized by the presence of any two of the three features: (i) polycysts in the ovaries, (ii) oligo/amenorrhea (absence or irregular menstrual cycle) and (iii) hyperandrogenism (2). Further, it co-exists with metabolic disturbances such as Insulin Resistance, obesity, dyslipidemia, diabetes, hypertension and many more (3, 4). Different pathways are involved in the pathogenesis of PCOS, and various symptomatic treatment approaches are available for the condition (5). Findings have shown that various lifestyle factors like unhealthy dietary patterns, smoking, alcohol consumption, sedentary lifestyle, etc., play a role in the aetiology of PCOS (6). Therefore, lifestyle modification (e.g., exercise, yoga, and a healthy diet) is one of the most relevant types of treatment that is recommended as the primary course of action (7–9).

“Yoga” is an age-old Indian practice that aims to balance the body, mind, and spirit (10, 11). Practising yoga regularly can restore balance and harmony within the whole body, including the reproductive system (12). Gonçalves et al. (13) showed that women with endometriosis, who practice yoga, reported lower levels of chronic pelvic discomfort and improved quality of life (13). It has been demonstrated that yoga, when used alone or in conjunction with other treatments, can effectively manage lifestyle diseases like diabetes, PCOS, and other reproductive disorders (14, 15). Another study reported a significant decrease in serum testosterone levels among women with PCOS after two months of walking and yoga (16). Further, people usually find yoga appealing due to its accessibility, low risk of adverse effects, and capacity to be tailored to each person's needs. Therefore, yoga must be explored as a complementary therapy for the management of PCOS.

The three aspects of yoga most frequently used for their health-promoting effects are asanas, pranayama, and meditation (17). *Asanas, or physical postures,* intended to support spiritual development, mental acuity, and bodily well-being (18). *Pranayama,* or yogic breathing techniques, can be done independently, or in combination with asanas to enhance its effects (19, 20). Studies have shown the benefits of pranayama on the physiological and psychological aspects of patients suffering from various diseases, including premenstrual syndrome (21, 22). *Mediation techniques* often play a significant role in enhancing an individual's mental health and overall well-being (23, 24). A study has shown that mindfulness-based treatments are beneficial for improving the mental health of infertile women (25, 26).

However, despite growing evidence supporting the role of lifestyle interventions and yoga in the management of PCOS, the findings across studies remain heterogeneous. A comprehensive synthesis of the available literature is therefore essential to clarify the therapeutic potential of yoga-based interventions and evaluate their impact on women with PCOS. In this context, this review aims to systematically synthesize existing evidence on yoga-based interventions for PCOS and identify gaps to guide clinical practice and future research.

## 2 Methodology

### 2.1 Prospero registration

The present study complies with the standard of PRISMA-2020 guidelines (Preferred Reporting Items for Systematic Review and Meta-analysis). PROSPERO registration ID for the study is CRD420261286708.

### 2.2 PIOS criteria

The following PIOS terms were used for this systematic review.

#### 2.2.1 Population (P)

Women diagnosed with PCOS based on any of the recognized diagnostic criteria (e.g., Rotterdam, NIH, or Androgen Excess Society criteria).

#### 2.2.2 Intervention (I)

The eligible interventions included yoga asana, meditation or mindfulness-based therapy.

#### 2.2.3 Outcomes (O)

Outcome measures include body composition (BMI; Body mass index, weight loss, waist-to-hip ratio, and waist circumference etc.), biochemical markers (lipid profile, triglycerides, LDL; Low-Density Lipoprotein, HDL; High Density Lipoprotein, blood glucose, insulin resistance, HOMA IR; Homeostatic Model Assessment of Insulin Resistance), or hormonal profiles (testosterone, estrogen, LH; Luteinizing Hormone, FSH; Follicle Stimulating Hormone, AMH; Anti Mullerian Hormone, SHBG; Sex Hormone-Binding Globulin), or psychological issues (stress, depression, quality of life, body Image etc.).

#### 2.2.4 Study design (S)

Randomized controlled trial (RCT).

Language: English only.

### 2.3 Inclusion and exclusion criteria for studies

The eligibility criteria for the selection of studies for inclusion in this systematic review are formulated as per the PIOS approach.

#### 2.3.1 Inclusion criteria

RCTs that examined the effects of any type of yoga interventions in PCOS women with relevant outcomes. Further, studies must feature yoga intervention conducted for a minimum duration of four weeks, with clear details on intervention (asanas, meditation or mindfulness-based), duration, etc. Only studies written in the English language were included.

#### 2.3.2 Exclusion criteria

Non-randomized studies, review articles, systematic reviews, case reports, conference abstracts, editorials, book chapters and letters to the editor were excluded. Studies without a comparator group or those that failed to report relevant outcomes were excluded. Trials involving participants with co-morbid conditions such as metabolic syndromes unrelated to PCOS, endocrine disorders, or other systemic illnesses that could confound the results were also excluded. Animal studies and *in vitro* research will be excluded. Studies conducted for less than four weeks will also be excluded from the review.

### 2.4 Data sources and search strategy

An extensive literature search in three databases, i.e., PubMed, Web of Science and Scopus, was performed on 17.1.2025. The search strategy included the following keywords along with their medical subject headings (MeSH terms) (Supplementary file S1).

### 2.5 Screening of studies and data extraction

The studies retrieved from all the databases were pooled and managed using blinded screening software RAYYAN (https://new.rayyan.ai/). Duplicates were removed manually by one author based on similarities in title, author, journal and year of publication, etc. Screening was done by two authors (RG and PM) independently in two-steps process, first by title and abstract and then by full text. Any discrepancies were resolved either through mutual discussion or by consulting the third author to ensure consistency and accuracy. Two authors performed data extraction. Data extraction sheet included study details such as author, publication year, country, study design and duration, participant characteristics, PCOS diagnostic criteria, sample size, intervention details, outcome measured, major findings and any adverse effects.

### 2.6 Risk of bias (quality) assessment

The risk of bias of all selected studies was assessed using the Cochrane Risk of Bias version 2.0 (RoB 2) (27). This tool analyzed each RCT in five domains: “bias in the randomization process, deviations from intended interventions, missing outcome data, outcome measurement, and selection of reported results”.

### 2.7 Meta-analysis

Initially, meta-analysis was planned for the included studies. However, we have received a limited number of studies. Further, these studies were heterogeneous in terms of the type of intervention, PCOS subjects recruited, duration of intervention, outcome measured, etc. Therefore, we could not perform a meta-analysis.

## 3 Result and discussion

### 3.1 Study selection

**Figure 1:**
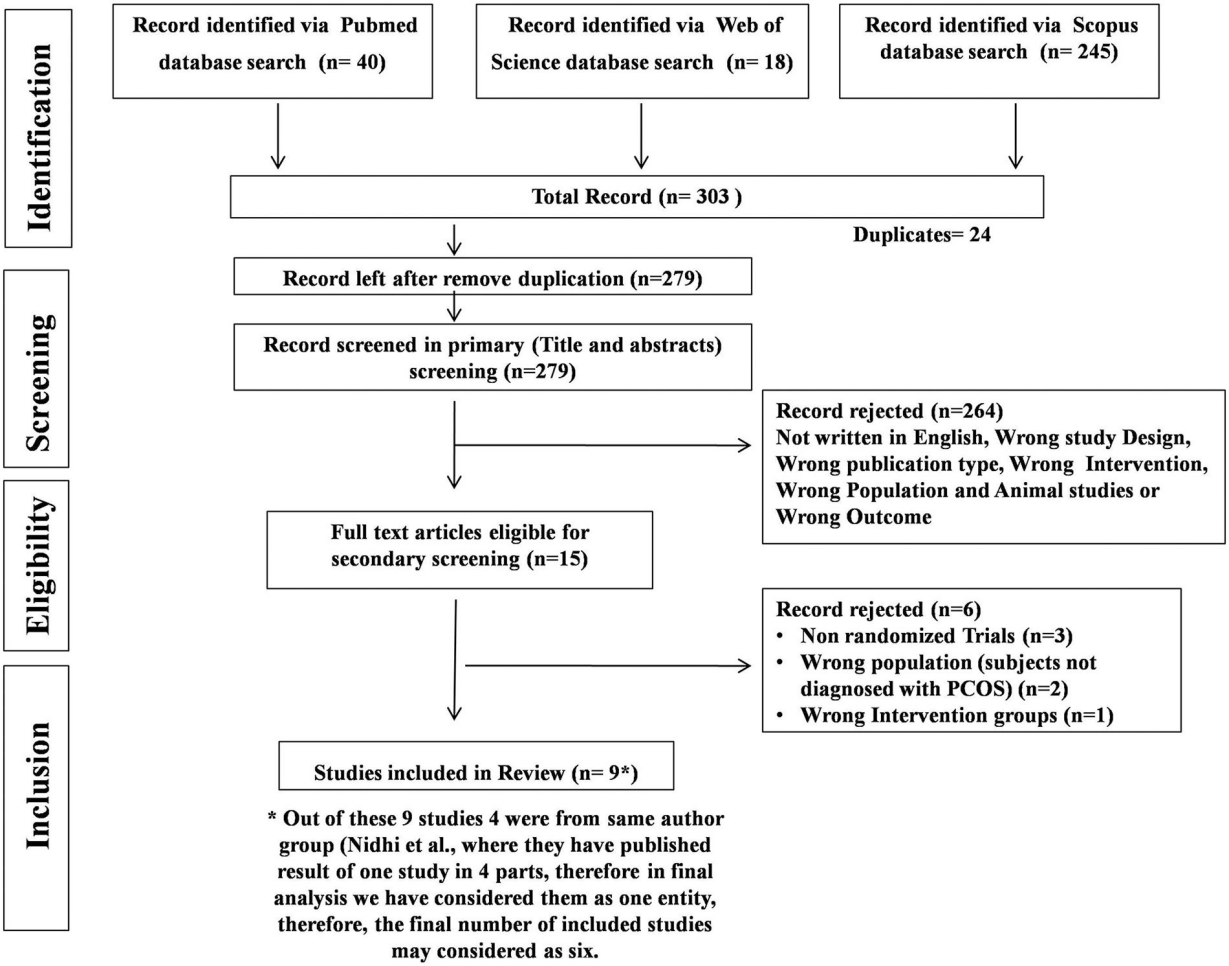
Prisma chart showing the screening and selection process.

We have received a total of 303 studies (MEDLINE 40, Scopus 245, Web of Science 18) out of which 44 studies were found to be duplicates. After removing duplicates, 279 studies were primarily screened by title and abstract. After primary screening, 15 articles were selected, and their full text was retrieved. After full-text screening, 9 articles were finally included in the study (Figure 1, Supplementary File S1 and S2). However, four of these studies were from the same author group Nidhi et al., where they have published the results of one study in four manuscripts. In the final analysis, we have considered them as one entity; therefore, the final number of included studies was six. Additionally in the study characteristic table (Table 1) of included studies the significant differences between the group (intervention vs. control) and within-group changes (post-intervention vs. baseline) are now clearly distinguished and denoted by * and #, respectively.

**Table 1:** Characteristic Table of studies showing effect of Yoga intervention of PCOS women.

S. No.	Author and year	Country	Study design and duration	Patient characteristics	Diagnostic criteria	Sample size		Details of intervention		Duration		Outcome measured	Outcomes in which significant change observed	Effect size of significantly changed outcome (mean difference = post-pre)		Adverse effect	Reference
						I	C	I	C	Per Day/Week	Total			I	C		
1	Mohseni et al. 2021	Tehran, Iran	RCT (Mar 2016–Nov 2016)	PCOS women undergoing infertility treatment	Rotterdam criteria	30	31	Exercises to reduce physical stress (25 min),	Routine care	90 min per session, daily (twice a week session were monitored by instructor at hospital gym)	6 weeks	Anthropometric parameter	*Hirsutism (mFG score)	*–1.74	*–0.29	Not Reported	(28)
								Asana exercises: (45 min), (makarasana, bhujangasana, dharmikasana, paschimottanasana; sardulasana; bhadrasana; matsyedrasana; savasana					*HC	*–7.17 cm	*–3.22 cm		
								Deep relaxation exercises (20 min)					*AC	*–6.60 cm	*–3.42 cm		
2	Patel et al. 2020	Erie County, Pennsylvania	RCT (Oct 2016–Jan 2017)	PCOS women Age: 23–42 years	Rotterdam criteria	13	9	Body awareness through a mental body scan	No Intervention	1 hour (Thrice a week with the instructor at wellness centre)	8 and 12 weeks	Endocrine, cardiometabolic, psychological parameters	^#^Free Testosterone	^#^−1.72 pg/mL	^#^–0.03 pg/mL	Not Reported	(16)
								Pranayama exercises (3-part yogic breath: ujjayi breath, alternate nostril breathing, and breath of fire)					^#^Adiponectin	^#^−1.80 µg/mL	^#^−0.90 µg/mL		
								Vinyasa flow yoga					^#^Dehydroepiandrosterone	^#^−43.10 ng/dL	^#^−14.40 ng/dL		
								Restorative yoga asanasMeditation.					^#^Anxiety (BAI Score)	^#^−3.10	^#^−1.10		
													^#^Depression (BDI-II Score)	^#^−8.75	^#^−6.00		
3	Nidhi et al. 2012a, 2012b, 2013 a and 2013b	Andhra Pradesh, India	RCT (Dec 2009–Jan 2011)	Adolescent girls with PCOS	Rotterdam criteria	37	35	Yoga Intervention	Matching set of physical 0exercise	60 min, daily (under the supervision of trained instructors at designated hall)	12 weeks	Quality of Life	*Emotional disturbances score	*–2.00	*–0.89	Not Reported	(29)
				Age: 15–18 years				Lecture (8 min),	Lecture (15 min),								
								Surya Namaskar (10 min)	Brisk walk (15 min),				*Body hair score	*–1.31 score	*–0.34 score		
								Yoga Asanas (Prone, Standing, Supine, Sitting) (12 min),	Physical Exercise (Prone, Standing, Supine, Sitting) (12 min)				*Weight perception Score	*–1.52	*–0.52		
								Guided relaxation (10 min),	Supine rest (10 min)				*Menstrual problems Score	*–2.35	*–1.13		
								Pranayama (10 min) Meditation (10 min)	Normal breathing (10 min)			Endocrine Parameters	*mFG score	*–1.13	*0.06		(30)
													*LH	*–4.10 IU/L	*3.00 IU/L		
													*LH/FSH ratio	*–1.17	*0.49		
													*Total testosterone	*–6.00 ng/dL	*2.61 ng/dL		
													*AMH	*–2.52 ng/mL	*–0.49 ng/mL		
													*Menstrual frequency	*+0.89 cycles/month	*+0.48 cycles/month		
												Anxiety symptoms	*Anxiety score	*–14.97	*–7.43		(31)
												Glucose metabolism and blood lipid levels	*Fasting insulin	*–9.04 µIU/mL	*11.09 µIU/mL		(32)
													*FBS	*–0.24 mg/dL	*0.04 mg/dL		
													*HOMA-IR	*–0.38	*0.28		
													*TG	*−0.14 mmol/L	*–0.07 mmol/L		
													*TC	*–0.24 mmol/L	*–0.08 mmol/L		
													*LDL	*–0.21 mmol/L	*–0.08 mmol/L		
													*VLDL	*–0.06 mmol/L	*–0.03 mmol/L		
													*TC/HDL ratio	*–0.33	*0.18		
Yoga Intervention (Meditation and Mindfulness Based Therapy)																	
4	Bafghi et al. 2024	Kerman, Iran	Single Blind RCT (Aug 2020–Jan 2021)	PCOS Women	Rotterdam criteria	30	30	Mindfulness-based art therapy sessions	No Intervention	90–120 min per session, twice a week	4 weeks	Body image scores	*Body image score	*+28.10	*−9.42	Not Reported	(33)
				Age:18–45 years				Mindful Body Scan Meditation,				Multidimensional Body-Self Relations					
								Embodied Breath Meditation,									
								Imagining Self-Care Based on Self-Image and Mindful Eating Exercise etc.				Questionnaire (MBSRQ)					
5	Young et al. 2021	Central Texas	Pilot RCT (Summers 2018–Apr 2019)	PCOS women	Not Mentioned	20	17	Mindfulness training program with health education in 4 key areas of self-management and health promotion: (1) medication adherence, (2) nutrition, (3) physical activity, and (4) sleep	No Intervention	Five 60- to 75-min consecutive weekly sessions,	5 weeks	Psychological distress, mindfulness, physical activity strategies, nutrition, and exercise self-efficacy	*Nutrition Self-efficacy score	*+5.70	*+1.00	Not Reported	(34)
				Age: 14–24 years						2 formal mindfulness exercises and review of homework practice followed by group discussions			*Physical activity strategies score	*+0.40	*0		
													*Physical activity self-efficacy score	*+0.30	*0		
6	Stefanaki et al. 2015	Athens, Greece	RCT (Nov 2012–May 2013)	PCOS women	Rotterdam criteria	23	15	Mindfulness breathing exercises and Diaphragmatic breathing exercises	No Intervention	30 min, daily	8 weeks	Stress, anxiety,	DASS21 Score			No Adverse effects reported in either group	(35)
				Age: 15–40 years								Depression, quality of life (DASS21, PSS-	*Depression	*–13.66	*–0.90		
												14, PCOSQ, Daily Life and General Life Satisfaction Questionnaires) and Salivary cortisol	*Stress	*–6.96	*+7.66		
													*Anxiety	*−4.00	*+3.07		
													*Salivary cortisol				
													At rest, wake up time	*–0.17 µg/dL	*+1.21 µg/dL		
													30 mins after wake up, at rest	*–1.16 µg/dL	*+0.12 µg/dL		
													Daily life questionnaire				
													*Life Satisfaction score	*+2.54	*–43.49		
													*General satisfaction score	*+1.95	*–0.13		

Sample size indicates the number of patients who completed the full interventions and included in final analysis. This does not includes the number of patients who drop out or lost to follow up from the study.

*Significant change between the groups (Intervention Vs Control) (In all studies except Patel et al., 2020 outcomes in which significant inter-group changes reported were mentioned in the table.

# Significant change within the group only (Post intervention Vs Baseline) (In Patel et al., 2020, as inter-group significant changes were not reported in any o the outcomes).

AC, abdominal circumference; AMH, anti-mullerian hormone; BAI, beck anxiety inventory score; BDI, beck depression inventory score; C, control group; DASS21, depression, anxiety, stress scales questionnaire; FBG, fasting blood glucose; FSH, follicle stimulating hormone; HC, hip circumference; HOMA_ IR, homeostatic model assessment of insulin resistance; I, intervention group, LDL, Low-density lipoprotein; LH, luteinizing hormone; MBSRQ, multidimensional body-self relations questionnaire; mFG score, modified ferriman-gallwey score; PCOSQ, PCOS health-related quality of life questionnaire; PSS-14, perceived stress scale questionnaire; RCT, randomized controlled trial; TC, total cholesterol; TG, triglycerides; VLDL, very low-density lipoprotein.

### 3.2 Description and heterogeneity of included studies

Although a meta-analysis was initially planned, it was not performed due to substantial heterogeneity across included studies. Clinical heterogeneity was evident in participant characteristics (e.g., adolescents, infertile women, and adult PCOS populations), while methodological heterogeneity existed in intervention types (yoga asanas, pranayama, and mindfulness-based therapies), duration (4–12 weeks), and study designs.

Among these 3 RCTs evaluated interventions combining various yoga asanas (physical postures) and Pranayama techniques (breathing exercises), while the remaining 3 RCTs employed meditation and mindfulness-based approaches (Table 1). All studies applied the Rotterdam criteria, except one, which did not specify the criteria, but reported only clinical diagnosis. The trials were conducted between 2012 and 2021, with no eligible study published thereafter. All included studies had small sample sizes, ranging from 20 to 70 participants, and were single-center trials. In each study, daily session durations were 60–120 min, with two or three times a week contact with the instructor. The total intervention duration for yoga-based studies ranged from 6 to 12 weeks, whereas mindfulness-based interventions lasted 4 to 8 weeks. The studies also exhibited substantial heterogeneity in participant characteristics. One trial included only adolescent girls with PCOS, another enrolled only infertile women with PCOS, and the remaining four recruited adult women with PCOS, but differed in age ranges and outcome measures. Owing to this clinical and methodological heterogeneity, a meta-analysis was not feasible, and the findings were synthesized narratively.

### 3.3 Outcomes

#### 3.3.1 Metabolic and anthropometric outcomes

Results from the included studies have shown that structured yoga interventions help in significantly improving glucose levels, insulin, HOMA-IR, lipid profile and adiponectin levels in women with PCOS (16, 32). Further included studies have also shown that Yoga intervention also resulted in significant reduction in anthropometric parameters such BMI, weight, WC, HC, Hirsutism score etc (28, 29). Findings from a recent pilot study on 35 PCOS women also showed that practicing yoga exercise (postures, breath work, and kriya) for six days a week for 12 weeks helped in decreasing fasting blood sugar and in improving insulin resistance (36). Banna et al. (2024) evaluated the effect of yoga compared to its combination with a Mediterranean diet rich in probiotics. They demonstrated that both groups showed significant improvement in weight, BMI, FBS, serum insulin and insulin resistance, with the combination group performing better, establishing yoga as a complementary therapy (37). A meta-analysis by Verma et al. (38) showed that yoga intervention decreases fasting blood glucose (MD .22 mmol/L, 95% CI .44–.01), fasting insulin (MD 28.21 pmol/L, 95% CI 43.79–12.63), and HOMA-IR (MD.86, 95% CI 1.29–.43) in PCOS women, but with low-grade evidence (38).

#### 3.3.2 Endocrine and menstrual health outcomes

Research has shown that practising specific yoga asanas, such as Ardha-Matsyendrasana (half-twist pose), Dhanurasana (bow pose), Vakrasana (twisted pose), Matsyendrasana (half-spinal twist), and Halasana (plough pose), along with meditation, helps in stimulating pancreatic or hormonal secretions in healthy individuals (39, 40). Findings from our included studies also revealed that yoga helps in reducing testosterone, LH, LH/FSH, AMH levels and improving menstrual cyclicity (16, 30). Meta-analysis by Verma et al. (38) also reported that yoga intervention can significantly decrease menstrual irregularity (MD .41, 95% CI .74–.08), clinical hyperandrogenism (MD .70, 95% CI 1.15–.26) in PCOS women (38). In another non-randomised clinical trial with infertile PCOS women, it was shown that structured yoga intervention (90 min daily for 12 weeks) resulted in improved metabolic health, menstrual health, ultrasound parameters and pregnancy rate (41).

One of the hallmarks of PCOS is the derangement of the hypothalamic-pituitary-ovarian (HPO) axis, that results in impaired gonadotropin secretion, higher LH/FSH ratio, causing anovulation and menstrual disturbances (42, 43). Evidence has suggested that the practice of yoga, along with naturopathy, can be effective for women with PCOS, to upregulate and balance the hypothalamic-pituitary-adrenal (HPA) axis, and improving LH and FSH secretion patterns and support ovulatory cycles (44). In a RCT study on 120 obese teenagers with PCOS, the intervention group (naturopathy and yoga interventions) showed a significant decline in testosterone and improvement in lipid profile as compared to the control group (45). In another study (case series of three PCOS women with dysmenorrhea), a regular yoga practice, naturopathy, and dietary changes are beneficial for the management of dysmenorrhea (46). In addition to the above factors oxidative stress also plays an important role in disrupting hormonal balance in PCOS women (47). Results have shown that structured yoga intervention (yoga therapy including asanas, pranayama and meditation) in Women with PCOS for 12-week showed a decline in, lipid peroxidation, inflammatory, oxidative stress markers, and an improvement in telomere length (48).

#### 3.3.3 Psychological outcome

PCOS does not only affect the patient physically but mentally as well due to issues like obesity, body image, infertility etc. Deep breathing, mindfulness, and meditation are examples of yoga techniques that stimulate the parasympathetic nervous system, which helps reduce the stress reaction. It encourages relaxation and lowers levels of stress related chemicals like cortisol which is a crucial stress indicator (49, 50). Results from five included studies showed yoga intervention resulted in significantly decrease in depression, stress, anxiety, body image, life satisfaction etc. in PCOS women (16, 28, 33, 34). It further enhances overall quality of life via enhancing Nutrition Self-efficacy score, Physical activity Self-efficacy score, and Life Satisfaction score in PCOS women (34, 35). A study showed that the customized Yoga Nidra treatments were successful in reducing the physiological stress response in PCOS-affected individuals (51). Further in a quasi-experimental study on 60 women with PCOS, the mindfulness based sessions result significant reduction in worries (worries related to mental complications, interpersonal problems, non-pregnancy physical complications, pregnancy complications, sexual complications, and religious issues) in the intervention group (52).

There are many biological mechanisms that may elucidate the observed advantages of yoga in PCOS. Firstly, yoga practices have been linked to increased insulin sensitivity, possibly via improved glucose metabolism and reduced sympathetic overactivity (21, 38, 53). Second, way is yoga and mindfulness-based interventions may modulate the hypothalamic–pituitary–adrenal (HPA) axis, resulting in decreased cortisol levels and enhanced stress response (49, 50). Since chronic stress and dysregulation of the HPA axis lead to insulin resistance and hyperandrogenism in PCOS, these effects may be clinically relevant. Lastly, practising yoga might also affect the hypothalamic pituitary ovarian (HPO) axis, which could help restore hormonal balance and make menstrual function better (42, 44). These mechanisms collectively offer a potential physiological foundation for the observed improvements; however, additional mechanistic investigations are required to validate these pathways.

### 3.4 Risk of bias

**Figure 2:**
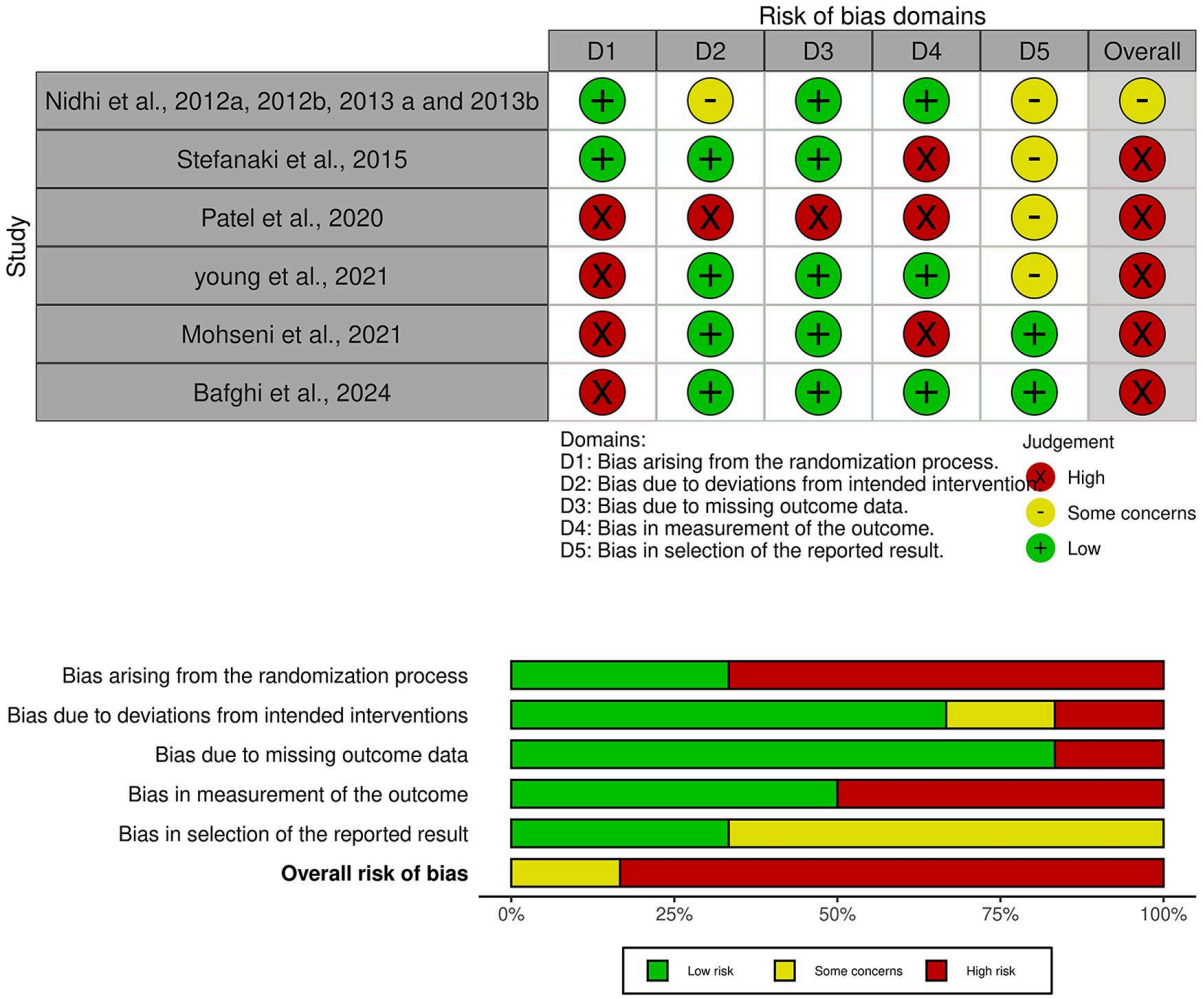
**(A)** RoB traffic light plot for individual studies included in the systematic review. Cochrane risk-of-bias tool was used to assess the six types of potential bias (selection, performance, detection, attrition, reporting, and other biases) in included studies, Each domain and study was rated each as low bias (+) (green color), high bias (−) (red color), or unclear bias (?) (yellow color) **(B)** Risk of bias summary Plot for the included studies.

The risk of bias assessment indicated that most included studies were at overall high risk of bias (Figure 2). A major concern across studies was bias arising from the randomization process (Domain 1), where several trials showed inadequate or unclear allocation concealment. Additionally, bias in outcome measurement (Domain 4) was observed, likely due to the lack of blinding in behavioral interventions such as yoga. While some studies demonstrated low risk in domains related to missing data and reporting, the cumulative assessment resulted in high overall risk for all included studies. These findings highlight the need for more rigorously designed trials in this field.

## 4 Limitations of the study

This study has several important limitations. First, there was substantial heterogeneity across the included interventions with respect to duration, population characteristics, and type of yoga and mindfulness based intervention protocols. This variability limits the ability to draw firm conclusions and prevents identification of the specific components responsible for observed effects. Second is most included studies also had small sample sizes, and single centric limiting the generalizability of the findings to broader populations. Additionally, follow-up periods were short, with the longest intervention lasting only three months, as a result, there is insufficient evidence regarding the long-term clinical outcomes and benefits. Further five out of six studies have not assessed or reported about any adverse effects of the intervention. Finally, the lack of standardized yoga protocols makes clinical implementation challenging.

## 5 Future perspective

Future randomized controlled trials on yoga in PCOS should be adequately powered to generate clinically meaningful evidence. Based on the effect sizes reported in published trials, the minimum required sample size calculated at a significance level of 0.02 and 90% power is approximately 86 participants (43 per group) for studies with similar objectives. Priority should be given to clinically meaningful and reproducible endpoints such as insulin resistance (HOMA-IR), menstrual cycle, androgen levels, and quality of life measures. Before widespread clinical implementation, there is a clear need to develop and validate a standardized yoga protocol defining the type of asanas, pranayama, meditation components, session duration, frequency, and minimum intervention length. In order to differentiate the effects of yoga from those of general physical activity or psychosocial support, future research should also incorporate suitable comparators. Long-term monitoring is also necessary to assess the benefits' durability. Until such evidence is available, yoga may be recommended only as a complementary lifestyle intervention alongside standard medical care for PCOS.

## 6 Conclusion

The current study represents Yoga may serve as a promising complementary, non-pharmacological approach for the management of PCOS, with potential benefits across metabolic, endocrine, menstrual, and psychological outcomes. However, the current evidence is limited by a small number of studies, small sample sizes, short intervention durations, and substantial heterogeneity in interventions and outcome measures. Therefore, well-designed, adequately powered randomized controlled trials with standardized yoga protocols and longer follow-up are needed to establish the efficacy and long-term benefits of yoga in PCOS management.

## Data Availability

The original contributions presented in the study are included in the article/Supplementary Material, further inquiries can be directed to the corresponding authors.
