# Supplementary material for: Yoga as a complementary intervention for polycystic ovary Syndrome management: a systematic review
Source: Front Reprod Health. 2026 Jun 3;8:1753608. doi: 10.3389/frph.2026.1753608 (PMC13272381; doi:10.3389/frph.2026.1753608)
Supplement: Supplementary file 1 [file Supplementaryfile1.docx]

**Supplementary Files**

**Supplementary File 1 : Search Strategy**

|  | **Keywords** | **PubMed** | **Scopus** | **Web of Science** |
| --- | --- | --- | --- | --- |
| *Population* #1 | “Polycystic Ovary Syndrome” OR PCOS OR “Polycystic Ovarian Syndrome” OR PCOD OR “Polycystic Ovary Disease” OR “Stein-Leventhal Syndrome” | 26,977 | 116,791 | 28,972 |
| *Intervention #*2 | Yoga OR Asana OR Meditation OR Pranayama OR “Mindfulness-Based Interventions” OR “Yoga Therapy” OR “Breathing Exercises” | 23,995 | 231,064 | 25,641 |
| *Final Search* #3 | #1 AND #2 | **40** | 1,065 | 35 |
| *Filters* | Limit to: Document Type (Article), Language (English), Open Access only  Limit to: Document Type (Article) | -  - | **245**  - | -  **18** |

**Search Results**

**PubMed:**

**
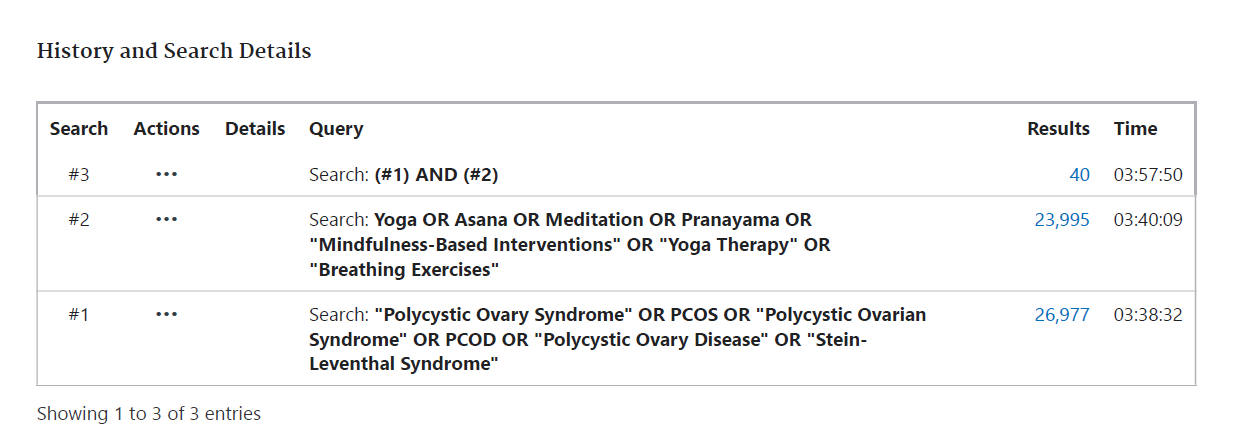
**

**Scopus:**

**
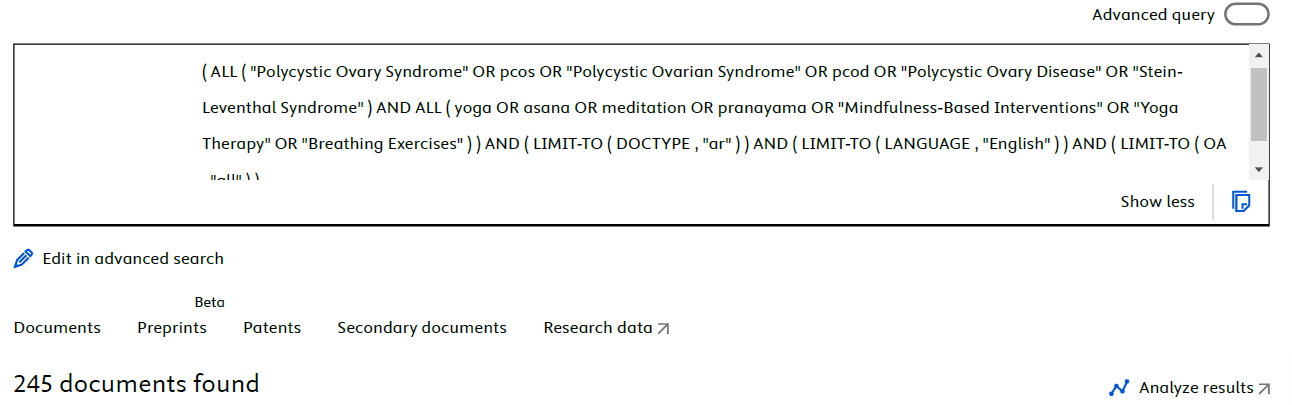
**

**Web of Science**

**
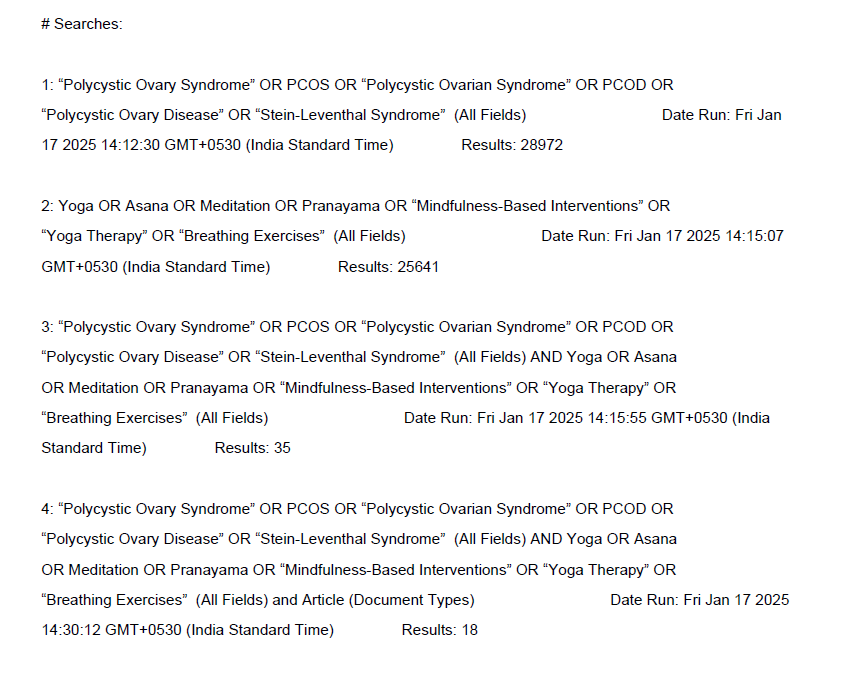
**

**Supplementary File : 2 List of Excluded Studies**

| **S. No.** | **Study** | **Exclusion Reason** |
| --- | --- | --- |
|  | Salajegheh, Z., Ahmadi, A., Shahrahmani, H., Jahani, Y., Alidousti, K., NasiriAmiri, F., &Salari, Z. (2023). Mindfulness-based stress reduction (MBSR) effects on the worries of women with poly cystic ovary syndrome (PCOS). *BMC psychiatry*, *23*(1), 185. | Non Randomized Trial (quasi-experimental design) |
|  | Dema, H., VidetičPaska, A., Kouter, K., Katrašnik, M., Jensterle, M., Janež, A., ...& Bon, J. (2023). Effects of mindfulness-based therapy on clinical symptoms and DNA methylation in patients with polycystic ovary syndrome and high metabolic risk. *Current issues in molecular biology*, *45*(4), 2717-2737. | Non Randomized Trial (quasi-experimental design) |
|  | Selvaraj, V., Vanitha, J., Dhanaraj, F. M., Sekar, P., &Babu, A. R. (2020). Impact of yoga and exercises on polycystic ovarian syndrome risk among adolescent schoolgirls in South India. *Health science reports*, *3*(4), e212. | Wrong Population (Adolescent girls of class 10 to 12 with NO PCOS Diagnosis) |
|  | Patil, A. D., Pathak, S. D., Kokate, P., Bhogal, R. S., Badave, A. S., Varadha, M., ... & Dalvi, P. D. (2023). Yoga intervention improves the metabolic parameters and quality of life among infertile women with polycystic ovary syndrome in indian population. *International journal of yoga*, *16*(2), 98-105. | Non Randomized Controlled Trial |
|  | Yang, N. Y., & Kim, S. D. (2016). Effects of a yoga program on menstrual cramps and menstrual distress in undergraduate students with primary dysmenorrhea: a single-blind, randomized controlled trial. *The Journal of Alternative and Complementary Medicine*, *22*(9), 732-738. | Wrong Population (Undergraduate Students with Menstrual Dysmennorhea) |
|  | ELBanna, M. M., Kamel, D. M., Shaaban, A., Botla, A. M., &Hamoda, R. E. (2024). Effect of Yoga Training and High Probiotic Food Supplements on Insulin-Resistance in Polycystic-Ovarian-Syndrome: A Randomized Controlled Trial. *International Journal of Kinesiology & Sports Science*, *12*(3), 52. | Compared (Mediterranean diet + Yoga) with Yoga alone. Tried to find the effect of adding Mediterranean diet with Yoga |
